# Some Prescriptions Discussed—III

**Published:** 1910-09-24

**Authors:** 


### THERAPEUTICS.

### SOME PRESCRIPTIONS DISCUSSED—III.

The following prescriptions, each of which is likely to be of service, are all found to yield good products from a dispensing point of view:—

Bromochloral Compound.

| | Parts. |
|---|---|
| Potassii bromidi ... | 27.00 |
| Chloral hydratis ... | 27.00 |
| Extracti cannabis indicæ | 0.22 |
| Extracti hyoscyami | 0.22 |
| Tincturæ aurantii... | 12.50 |
| Glycerini ... | 18.75 |
| Aquam ad ... | 100.00 |

This is a Scotch formulary, and it differs from the similar Codex preparation in containing 15 grains instead of 10 grains each of potassium bromide and of chloral hydrate in each fluid drachm. The product retains all the cannabis indica in solution, and it keeps well without forming any deposit.

Pulvis pro Gargarisma.

| | Parts. |
|---|---|
| Pulveris potassii chloratis | 12.50 |
| Sodii biboratis ... | 12.50 |
| Sodii chloridi ... | 25.00 |
| Sodii bicarbonatis ... | 50.00 |

The powder can be dissolved in a sufficiency of warm water, and used more or less dilute as may be required in different cases.

The outcome of numerous experiments made by a special committee, with a view to devising the most satisfactory antiseptic and emollient dusting powder, has been the following formulary, which is closely analogous to the ordinary triple powder:—

Pulvis Zinci et Amyli Compositus.

| | Parts. |
|---|---|
| Zinci oxidi ... | 25.00 |
| Pulveris Amyli ... | 25.00 |
| Pulveris acidi borici | 25.00 |
| Pulveris cretæ gallici | 25.00 |
| Olei rosæ geranii ... | 0.20 |

The ingredients should be in superfine powder, and when the mixing has been thoroughly completed, the product should be sifted through a No. 60 sieve.

The following sprays have been found to yield good products:—

Nebula Hyoscini Composita.

| | Parts. |
|---|---|
| Hyoscini hydrobromidi | 0.10 |
| Cocainæ ... | 0.90 |
| Atropinæ sulphatis | 0.05 |
| Glycerini ... | 25.00 |
| Liquorem thymolis compositum ad | 100.00 |
| Caramel q.s. to colour like sherry. | |

Nebula Mentholis Composita.

| | Parts. |
|---|---|
| Mentholi ... | 2.08 |
| Thymoli ... | 2.08 |
| Camphoræ ... | 2.08 |
| Phenoli ... | 2.08 |
| Paraffinum liquidum B.P. ad ... | 100.00 |

Nebula Thymolis Composita.

| | Parts. |
|---|---|
| Camphoræ ... | 2.00 |
| Mentholi ... | 2.00 |
| Thymoli ... | 0.50 |
| Eucalyptoli ... | 8.50 |
| Paraffinum liquidum B.P. ad ... | 100.00 |

As a liniment for local application in cases of painful joints, sciatica, myalgia, and the like, the following liniment of betul oil has been recommended as an excellent preparation:—

Linimentum Methyl-Salicylatis Compositum.

| | Parts. |
|---|---|
| Mentholi ... | 5.00 |
| Chloral hydratis ... | 5.00 |
| Spiritus rectificati ... | 10.00 |
| Tincturæ cannabis indicæ | 10.00 |
| Olei camphoræ essentialis | 20.00 |
| Methyl-salicylatic ad ... | 100.00 |

An ointment which has similar strong analgesic properties, for application over unbroken skin, is the following:—

Unguentum Methyl-salicylatis cum Mentholo.

| | Parts. |
|---|---|
| Methyl salicylatis ... | 50.00 |
| Mentholi ... | 10.00 |
| Eucalyptoli ... | 2.50 |
| Olei cajuputi ... | 2.50 |
| Ceræ albæ ... | 30.00 |
| Unguentum lanæ hydrosæ ad ... | 100.00 |

The last ingredient of the above prescription is a very useful ointment basis containing both paraffin and excess of wool fat, as follows:—

Unguentum Lanæ Hydrosæ.

| | |
|---|---|
| Adipis lanæ hydrosi / Paraffini mollis flavi } ... | aa |

and if preferred, the methyl-salicylate analgesic ointment may be made without other ingredients, as follows:—

Unguentum Methyl Salicylatis.

| | Parts. |
|---|---|
| Methyl salicylatis ... | 50.00 |
| Ceræ albæ ... | 25.00 |
| Unguenti lanæ hydrosæ ... | 25.00 |

We will conclude by giving two resorcin ointments that have been recommended by a Scotch committee of pharmacists:—

Unguentum Resorcini Compositum.

| | Parts. |
|---|---|
| Resorcini ... | 8.00 |
| Aquæ ... | 12.00 |
| Pulveris amyli ... | 20.00 |
| Zinci oxidi ... | 8.00 |
| Bismuthi ... | 8.00 |
| Olei cadini ... | 2.50 |
| Olei rusci ... | 2.50 |
| Unguentum lanæ anhydrosæ ad ... | 100.00 |

Unguentum Ichthyamolin Resorcino.

| | Parts. |
|---|---|
| Resorcini ... | 3.00 |
| Aquæ ... | 9.00 |
| Acidi salicylici ... | 3.00 |
| Beta-Naphtholi ... | 3.00 |
| Ichthyamoli ... | 9.00 |
| Sulphuris præcipitatis ... | 9.00 |
| Zinci oxidi ... | 9.00 |
| Pulveris amyli ... | 9.00 |
| Unguentum lanæ anhydrosæ ad ... | 100.00 |

Either of these ointments may be used in conditions such as psoriasis; the first being milder than the second is more applicable in the acuter stages. If it is desired, salicylic acid to the extent of 2 parts per 100 may be added to the first prescription, to make a preparation which is intermediate between the two in regard to its stimulating action.

